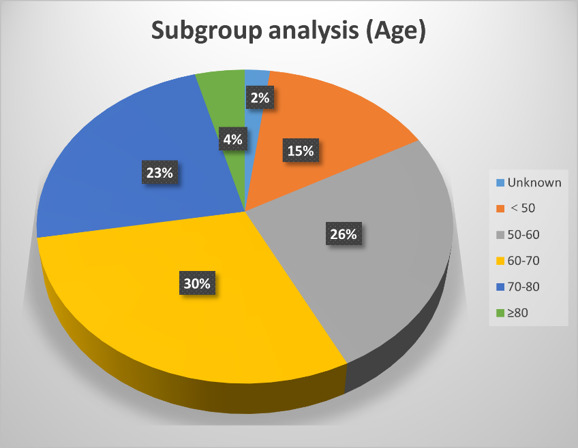# A giant peritoneal loose body: A case report and updated literature review and data synthesis

**DOI:** 10.1097/MD.0000000000045956

**Published:** 2025-12-12

**Authors:** Guo-Le Nie, Shicheng Chu, Song Geng, Hong Jiang, Hao Zhan

**Affiliations:** a Department of Colorectal Hernia Surgery, Binzhou Medical University Hospital, Binzhou, China; b The First School of Clinical Medicine of Binzhou Medical University, Binzhou, China.

**Keywords:** case report, CT, laparoscopic exploration, nodular fibrous pseudotumour, peritoneal loose body

## Abstract

We report an 80-year-old male with a nodular fibrous pseudotumor (NFP), a rare benign tumor typically linked to trauma, surgery, or inflammation. Unusually, it presented as a mobile peritoneal loose body (PLB) despite no relevant abdominal history. Imaging showed a well-defined oval mass (70 × 63 mm). Pathological examination confirmed NFP, showing hyalinized collagen and fibroblast proliferation without atypia. This is the first NFP presenting as a PLB at our institution. The patient underwent successful laparoscopic resection with no recurrence during follow-up. This case highlights that: NFP can mimic mobile intraperitoneal lesions, requiring distinction from malignancy; pathological confirmation is essential due to overlapping computed tomography features with metastases; and the PLB presentation expands the known etiological spectrum beyond classical triggers. NFP should be considered in the differential diagnosis of peritoneal nodules, especially in atypical scenarios.

## 1. Introduction

Nodular fibrous pseudotumor (NFP) is a rare benign tumor-like lesion predominantly associated with a history of abdominal surgery, trauma, or inflammation.^[1]^ As a distinct pathological subtype of peritoneal loose bodies (PLBs), NFP was analyzed in an abdominal cavity case herein, complemented by a literature review. This study aims to summarize its clinical and imaging manifestations, thereby enhancing clinicians’ recognition and diagnostic accuracy of this entity.

## 2. Case description

**Figure 1.**
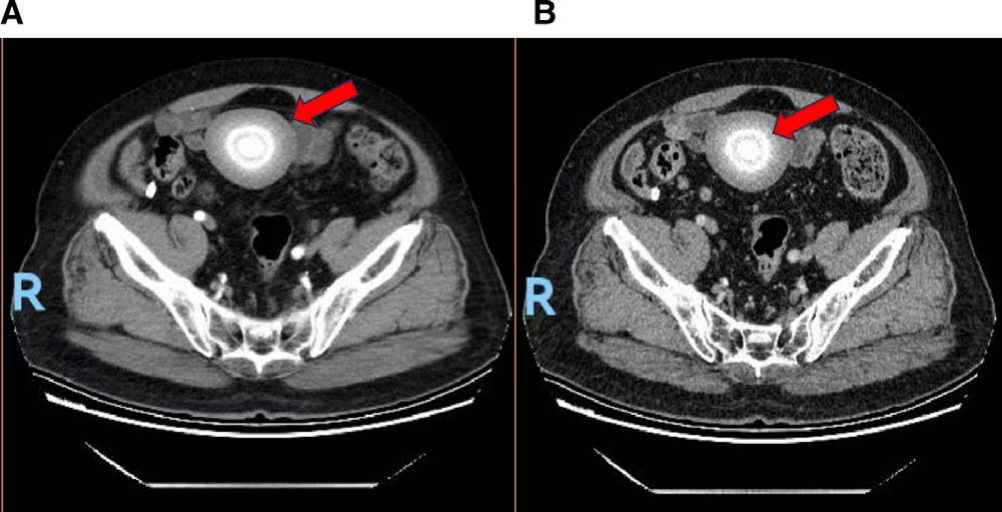
CT imaging of abdominal masses. (A) arterial phase; (B) venous phase. CT = computed tomography.

In October 2024, an 80-year-old male was referred to our department for evaluation of an incidentally discovered, mobile abdominal mass. He had no significant history of abdominal surgery, trauma, or intra-abdominal inflammation. Physical examination revealed a palpable, non-tender, and moderately mobile mass in the lower abdomen. An abdominal computed tomography (CT) scan demonstrated a well-defined, oval, soft-tissue density mass (70 × 63 mm in maximal diameter) located in the right anterior pelvic space, superior to the bladder, and enhanced scan showed no significant enhancement.. The lesion showed heterogeneous attenuation and characteristic internal dual-ring calcifications (Fig. 1).

Given the imaging findings, laparoscopic resection of the pelvic mass was performed. Diagnostic laparoscopy revealed normal abdominal organs and identified a white, firm mass in the pelvic cavity with a smooth surface and focal adhesions to adjacent tissues. The mass is only mildly adherent to the abdominal wall. Adhesion characteristics: avascular, soft collagen fiber bundles (2–3 mm thick). Following adhesiolysis and complete mobilization of the mass, the specimen was retrieved through a 10-cm lower abdominal incision prior to excision. The patient was discharged on postoperative day 7 without complications and remained recurrence-free at the last follow-up in April 2024.

## 3. Pathological examination

**Figure 2.**
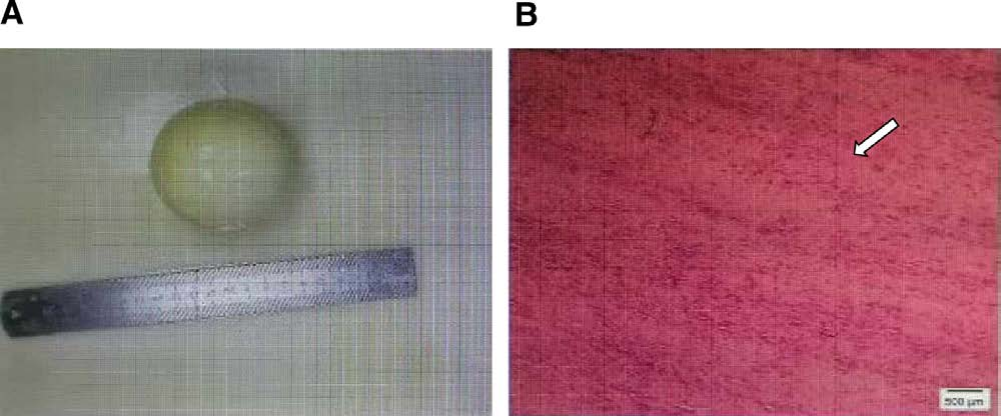
Pathological Examination. (A) A broad view of nodular fibrous pseudotumour. (B) HE staining results of nodular fibrous pseudotumour. The white arrow shows that densely arranged collagen bundles interspersed with scattered bland spindle cells (fibroblasts). Scale bar: 500 μm. HE = hematoxylin-eosin staining.

Gross examination revealed a gray-yellow, oval mass measuring 7.2 × 6.3 × 3.0 cm, featuring a smooth serosal surface and a firm, calcified core. Histologically, the lesion comprised densely arranged collagen bundles interspersed with scattered bland spindle cells (fibroblasts), accompanied by foci of hyalinization and dystrophic calcification. These features are diagnostic of NFP (Fig. 2).

## 4. Literature search strategy and selection criteria

Search strategy: we conducted a structured literature search in the electronic databases PubMed, Scopus, and Web of Science to identify relevant English-language case reports and series on PLBs. The search was performed from inception until March 2024. The key search terms were: (“peritoneal loose body” OR “peritoneal mouse” OR “peritoneal free body”) AND (“case report” OR “case series”). Inclusion and exclusion criteria: we included all published case reports and case series that provided unique clinical or pathological details of PLBs. Studies not in English, reviews without original case data, and conference abstracts with insufficient information were excluded. Systematic approach: systematic reviews and network meta-analyses can assist clinical decision makers in exploring clinically relevant questions,^[2–4]^ while our review is not a formal systematic review and meta-analysis, we adopted the PRISMA guidelines to enhance the transparency and rigor of our process. The literature search and selection were performed independently by 2 authors to minimize bias.

## 5. Discussion

### 5.1. Epidemiological and characteristics of PLBs

**Figure 3.**
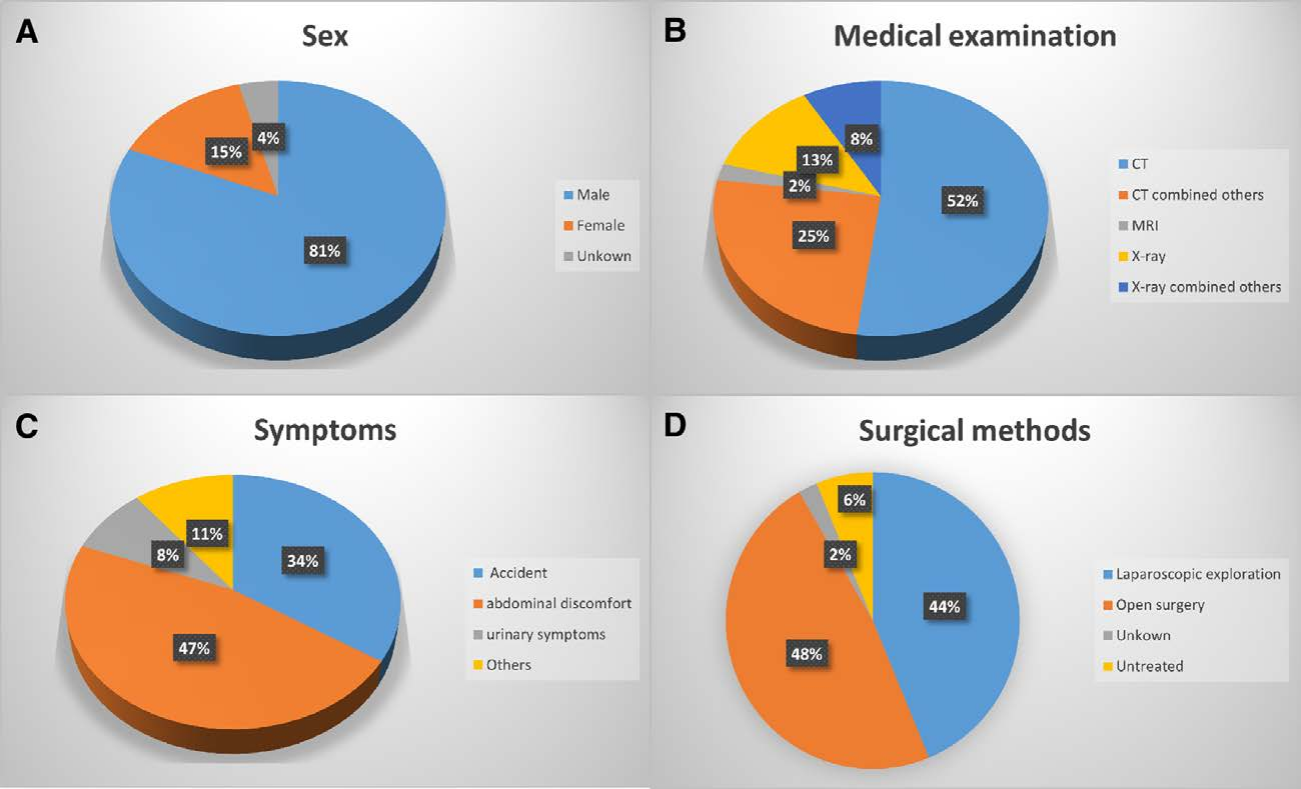
Percentage of PLBs by age, medical examination, symptoms, and surgical approach. (A) Percentage of distribution of age; (B) percentage of medical examination; (C) percentage of symptoms; and (D) percentage of surgical methods. PLBs = peritoneal loose bodies.

PLBs are predominantly small (0.5–2.5 cm), incidentally detected lesions, and remain asymptomatic in > 90% of cases.^[5]^ Their incidence demonstrates a predilection for elderly males, as evidenced by demographic analyses.^[6,7]^ Table 1 shows the characteristics of previous cases. The sources of some key data in this study. Table 2 synthesizes clinicopathological profiles of 47 published cases, highlighting this demographic bias and size distribution. Synthesis of published evidence indicates a significant male predominance in PLBs occurrence (Fig. 3A), with > 85% incidentally detected during imaging. Symptomatic presentations (Fig. 3C), primarily abdominal discomfort or urinary complaints, correlate strongly with lesion size > 3 cm and proximity to visceral organs. Age subgroup analysis showed that the high-incidence age groups for PLBs were 50–60 (26%), 60–70 (30%), and 70–80 (23%) years old (Fig. S1, Supplemental Digital Content, https://links.lww.com/MD/Q731)

**Table 1:** The raw data PLBs reported in the literature.

References	Year	Age (yr)	Gender	Size (mm)	Medical examination	Symptoms	Surgical methods
Zhang et al^[5]^	2015	51	M	50 × 40	Ultrasonography and CT	Incidental	Laparoscopic exploration
Kim et al^[6]^	2013	50	M	75 × 70 × 68	CT	Incidental	Laparoscopic exploration
Mohri et al^[7]^	2007	73	M	95 × 75	CT and MRI	Abdominal pain	Open surgery
Baert et al^[8]^	2019	53	M	55	CT	Abdominal pain and constipation	Laparoscopic exploration
Guo et al^[9]^	2019	49	M	55 × 50	CT	Abdominal pain	Open surgery
Rykovský and Michal^[10]^	2023	Unknown	Unknown	Unknown	CT angiography and PET/CT		Open surgery
Nanno et al^[11]^	2023	83	M	60	CT	Incidental	Laparoscopic exploration
Sang et al^[12]^	2024	67	M	45 × 43	CT	Recurrent urethral bleeding	Laparoscopic exploration
Matsubara et al^[13]^	2017	70	M	58	CT and MRI	Urinary frequency	Laparoscopic exploration
Teklewold et al^[14]^	2019	50	M	75 × 60 × 50	X-ray	Abdominal pain and abdominal obstruction	Open surgery
Wu et al^[15]^	2024	68	M	115 × 86 × 74	CT	Abdominal pain	Open surgery
Elsner et al^[16]^	2016	52	M	52	X-ray and CT	Proctitis	Laparoscopic exploration
Makineni et al^[17]^	2014	52	M	60	CT	Discomfort in the lower abdomen and irritative voiding symptoms	Open surgery
Mehammed et al^[18]^	2024	61	M	65	CT	Lower abdominal pain and irritative voiding symptoms	Open surgery
Ansari et al^[19]^	2022	50	M	70 × 60	ultrasonography and CT	Heaviness in the right lower abdomen	Open surgery
Cojocari and David^[20]^	2018	72	M	58 × 65	CT	Incidental	Open surgery
Oom et al^[21]^	2018	64	M	60 × 40 × 60	CT	Incidental	Open surgery
Huang et al^[22]^	2017	79	M	104 × 83 and 76 × 60	CT	Frequency of urination	Open surgery
Jang et al^[23]^	2012	60	M	45 × 45 × 30	CT and MRI	Incidental	Laparoscopic exploration
Obaid and Gehani^[24]^	2018	58	M	49 × 41	CT	Abdominal pain and hematuria	Laparoscopic exploration
Li et al^[25]^	2020	49	M	45 × 40 × 33	CT	Abdominal mass and urinary frequency	Laparoscopic exploration
Nomura et al^[26]^	2003	63	M	50	CT and MRI	Incidental	Laparoscopic exploration
Allopi et al^[27]^	2021	79	M	45	CT	Abdominal pain	Laparoscopic exploration
Sewkani et al^[28]^	2011	64	M	70 × 50	X-ray	Acute intestinal obstruction	Open surgery
Suganuma et al^[29]^	2014	35	F	60 × 50	MRI	Incidental	Laparoscopic exploration
Lee et al^[30]^	2017	61	F	60	CT	Abdominal pain	Laparoscopic exploration
Rosic et al^[31]^	2016	73	M	66 × 56 × 40	CT	Urinary symptoms	Laparoscopic exploration
Ohgitani et al^[32]^	2004	Unknown	Unknown	Unknown	CT and MRI	Accident	Unknown
Hedawoo and Wagh^[33]^	2010	65	M	95 × 86	CT	Abdominal pain	Open surgery
Dhoot et al^[34]^	2020	75	M	62 × 58	CT	Chronic intermittent abdominal discomfort with acute diarrhea and perianal pain	Laparoscopic exploration
Sahadev and Nagappa^[35]^	2014	52	M	70 × 60	ultrasonography and CT	Incidental	Laparoscopic exploration
Rubinkiewicz et al^[36]^	2014	70	F	200 × 100	X-ray and ultrasonography	Bowel obstruction	Open surgery
Murat and Gettman^[37]^	2004	47	M	35 × 28 × 25	CT	Pelvic pain	Laparoscopic exploration
Bhandarwar et al^[38]^	1996	65	M	90 × 80	X-ray	Acute retention of urine	Open surgery
Shepherd^[39]^	1951	79	M	70 × 55	X-ray	Acute retention of urine	Open surgery
Ghosh et al^[40]^	2006	63	M	58 × 45 × 37 and 52 × 45 × 37	X-ray and ultrasonography	Intestinal obstruction	Open surgery
Kao et al^[41]^	2010	69	F	40 × 30 × 23	X-ray and ultrasonography	Intestinal obstruction	Open surgery
Koga et al^[42]^	2010	33	F	30 × 20	X-ray	Infertility	Laparoscopic exploration
Gayer and Petrovitch^[43]^	2011	59	M	30	CT	Incidental	Untreated
Asabe et al^[44]^	2005	2 mo	F	30	CT	Urinary tract infection	Laparoscopic exploration
Nozu et al^[1]^	2012	67	M	40	CT	Incidental	Untreated
Burns and Rogers^[45]^	1969	33	F	18 × 13	X-ray	Incidental	Open surgery
Maekawa et al^[46]^	2013	58	M	20	CT	Incidental	Open surgery
Allam et al^[47]^	2013	77	M	17	PET-CT and CT	Abdominal pain	Untreated
Huang et al^[48]^	2011	55	M	–	X-ray and ultrasonography	Intestinal obstruction	Open surgery
Takada et al^[49]^	1998	79	M	70 × 60 and 70 × 60	CT and MRI	Incidental	Open surgery
Arwikar et al^[50]^	2022	65	M	15	CT	Vomiting and abdominal pain	Open surgery
Our center	2024	80	M	72	CT	Incidental	Laparoscopic exploration
Abstract	•Most PLBs are 0.5–2.5 cm in diameter and, therefore, most PLBs are asymptomatic. •PLBs occurred more often in male patients. •PLBs are found incidentally in most patients. • PLB patients usually have abdominal discomfort and urinary symptoms. •PLBs are usually diagnosed by CT. •The treatment of PLBs include mainly surgery and wait-and-see.						

CT = computed tomography, F = female, M = male, MRI = magnetic resonance imaging, PET = positron emission tomography, PLBs = peritoneal loose bodies.

**Table 2:** The summary of PLBs reported in the literature.

Characteristics	Value
Patients, n	48
Age, yr	61.71 ± 12.57
Sex, n (%)	
M	39 (81)
F	7 (15)
Unknown	2 (4)
Surgical approach, n (%)	
Laparoscopic exploration	21 (44)
Open surgery	23 (48)
Untreated	3 (6)
Unknow	1 (2)
Size, mm	45.56 ± 18.12
Medical examination, n (%)	
CT	25 (52)
CT combined others	12 (25)
MRI	1 (2)
X-ray	6 (13)
X-ray combined others	4 (8)

CT = computed tomography, F = female, M = male, MRI = magnetic resonance imaging, PLBs = peritoneal loose bodies.

At our institution, palpable mobile masses represented the chief symptom, attributable to large dimensions (>6 cm), peritoneal mobility, and absence of adhesions.

### 5.2. Symptom-driven management algorithm

**Figure 4.**
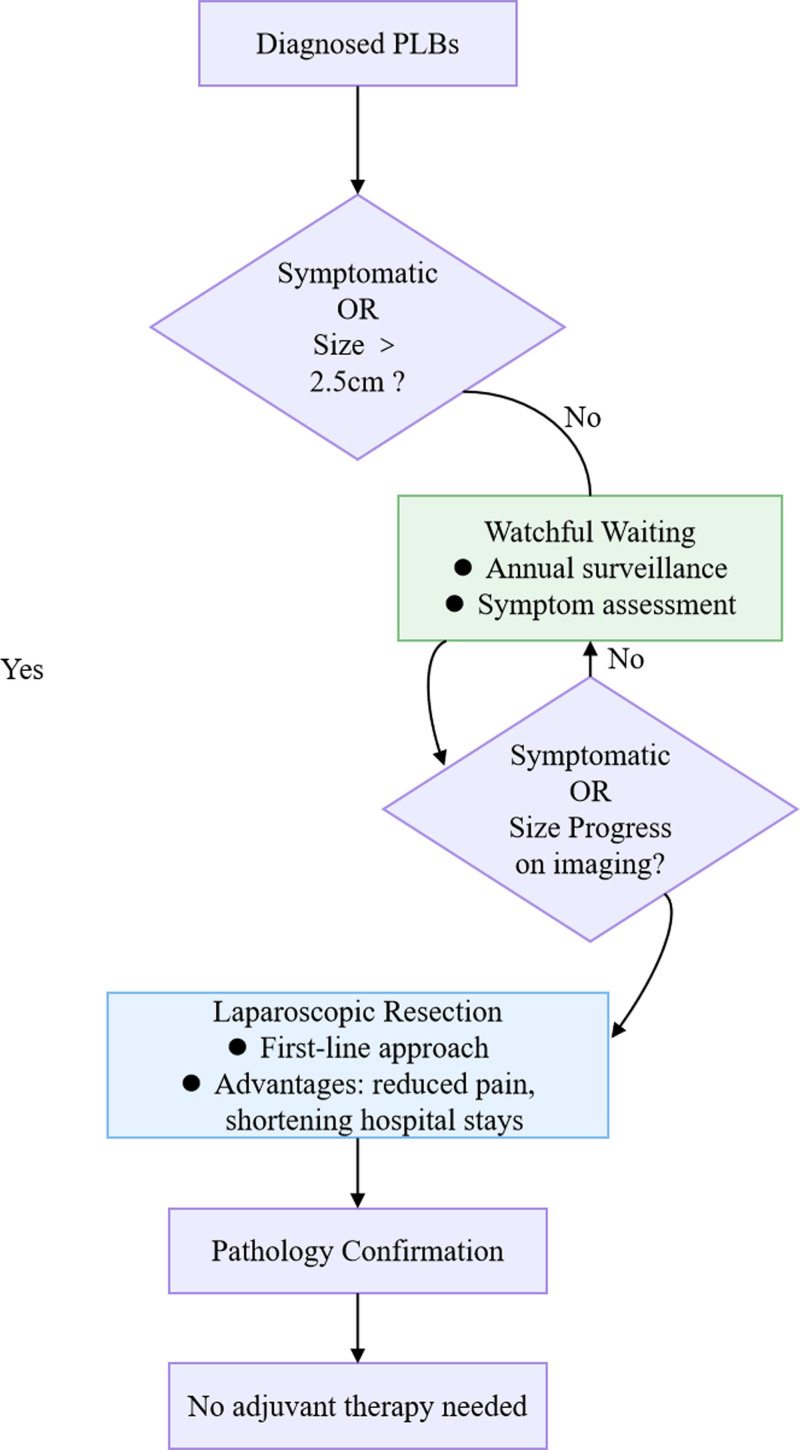
The management algorithm for PLBs. PLBs = peritoneal loose bodies.

Current approaches regarding the treatment of PLBs include mainly surgical intervention and watchful waiting (Fig. 3D). We propose a dual-pathway classification system based on clinical presentation:

Asymptomatic PLBs: for radiologically identified lesions ≤ 2.5 cm, watchful waiting represents a viable option after shared decision-making that incorporates patient preference and comorbidity profiles. Symptomatic PLBs: requires comprehensive radiological characterization (size/location/organ compression). Surgical intervention should be considered following informed consent. Figure 4 shows the management algorithm for PLBs.

### 5.3. Modality-directed intervention protocol

PLBs may be categorized by detection method: CT-diagnosed PLBs: management hinges on tripartite analysis of lesion size, anatomical relations, and patient-specific risk-benefit assessment. Intraoperatively discovered PLBs: resection decisions require real-time consultation with patient-designated healthcare proxies and documented institutional ethics committee protocols.

### 5.4. Pathogenetic mechanisms and molecular drivers

While peritoneal loose body (PLB) pathogenesis remains incompletely elucidated, 2 primary origins are hypothesized: appendices epiploicae,^[51]^ and autoerotic ovaries or uterus.^[29,30,36,45]^ Ischemia-induced fat necrosis promotes saponification/calcification.^[48,49]^ Subsequently, abnormal collagen deposition promotes its progressive enlargement.^[12]^ Studies have shown that PLBs are mainly composed of collagen and proteins and lack normal cellular structure. Proteomic analyses identify 42 collagen-associated proteins, with 3 key mediators: ASPN (induces calcification via collagen binding).^[52]^ Asporin (a protein known to promote calcification in orthopedic contexts) was identified in a PLB proteomic study, suggesting a role in calcification of these lesions. Laminin (scaffolds collagen network assembly). Laminin also plays a key role in collagen network formation.^[53]^ Cnnective tissue growth factor (CTGF)/COL5A2/COL1A1 (drive pathological collagen deposition). CTGF and various collagen isoforms such as COL5A2 and COL1A1 have important roles in collagen deposition and network formation.^[54–56]^ PLBs are more likely to originate from the omentum or mesenteric fat.^[46]^ Although representative images are lacking, in our center’s minimally invasive surgery patients, it can be seen that the omentum or mesenteric fat is about to detach in some patients. These parts of the omentum or mesenteric fat that were about to detach may be precursors to the formation of PLBs. The specific cause of the particular type of NFP that developed in this patient is unclear. When we reviewed the patient’s medical history, we found that he had no history of trauma, diverticulitis, or surgery. This is one of the reasons why this case is so unusual.

### 5.5. Diagnostic imaging hallmarks of PLBs

Past case reports show that CT remains the mainstream method for diagnosing PLBs (Fig. 3B). PLBs demonstrate characteristic multimodality imaging patterns: Ultrasound: well-circumscribed, round, hypoechoic mass; central hyperechoic focus with posterior acoustic shadowing; absence of Doppler flow; and probe-induced mobility during compression. X-ray: spherical, smooth-margined hyperdense opacity; and positional variability on serial imaging. CT: Circumscribed soft-tissue attenuation mass; pathognomonic central calcification (irregular/patchy/concentric); absence of contrast enhancement; migration sign on repositioning. Magnetic resonance imaging (MRI): isointense to skeletal muscle on T1/T2-weighted sequences; T1-hyperintense core (fatty component); and non-enhancement post-contrast.^[11]^

While ultrasound, X-ray, and CT typically provide definitive diagnosis, MRI enhances specificity in differentiating PLBs from pelvic malignancies (e.g., ovarian teratomas, metastatic deposits) by characterizing fat content and enhancement patterns. PET-CT may be considered when metabolic activity requires assessment to exclude malignant transformation.^[47]^ PLBs are usually diagnosed by CT or in combination with other investigations (ultrasound, etc) as reported in the previous literature (Fig. 3B).

### 5.6. Differential diagnosis of PLBs

NFP should be differentiated from the following diseases: solitary fibrous tumor, desmoid fibromatosis (Desmoid), calcifying fibroma (CFT), etc. NFP is typically a reactive/repair lesion, often associated with a history of surgery, foreign bodies, inflammation, or hernias. It is usually asymptomatic or presents with mild symptoms and is often discovered incidentally. Solitary fibrous tumor is typically a primary tumor with no specific precipitating factors. Its presentation is diverse, ranging from asymptomatic to compressive symptoms or paraneoplastic syndromes. Pathological features often include: often have a pseudocapsule; the cut surface is grayish-white and firm, may be accompanied by mucinous degeneration or hemorrhagic foci. Desmoid tumors may occur sporadically or be associated with familial adenomatous polyposis/Gardner syndrome. Symptoms or manifestations may include painful masses with local invasiveness. Pathological features often include a grayish-white or yellowish-brown cut surface, firm or fleshy texture. The etiology of CFT is currently unknown, and it is typically sporadic. Pathologically, it is typically grayish-white in color, with a gritty texture (calcification) commonly observed. Currently, there are few reports on the attenuation values of PLBs. According to our center and previous cases, for typical PLBs, the HU values of their outer surfaces are similar to those of soft tissue, while the HU values of their internal calcifications are similar to those of bone tissue.

The references involved in the comparisons are all directly marked in the corresponding tables (see Table 1).^[1,5–50]^

## 6. Conclusion

PLBs predominantly represent incidental radiological findings. Clinicians must include PLB in the differential diagnosis when encountering an isolated, oval intraperitoneal mass with characteristic calcifications on imaging. For symptomatic patients exhibiting urinary dysfunction or abdominal discomfort, prompt laparoscopic resection is strongly recommended as the gold-standard intervention, given its superior recovery profile and diagnostic-therapeutic duality. This approach optimizes both pathological confirmation and definitive management within a single procedure.

## Author contributions

**Conceptualization:** Guo-Le Nie, Hao Zhan.

**Data curation:** Guo-Le Nie, Shicheng Chu, Song Geng.

**Formal analysis:** Guo-Le Nie.

**Investigation:** Guo-Le Nie, Shicheng Chu, Song Geng.

**Methodology:** Guo-Le Nie.

**Project administration:** Guo-Le Nie, Hong Jiang, Hao Zhan.

**Resources:** Shicheng Chu.

**Supervision:** Hong Jiang, Hao Zhan.

**Visualization:** Guo-Le Nie.

**Writing – original draft:** Guo-Le Nie, Shicheng Chu, Song Geng, Hong Jiang, Hao Zhan.

**Writing – review & editing:** Guo-Le Nie, Shicheng Chu, Song Geng, Hong Jiang, Hao Zhan.

## Supplementary Material

**Figure:**